# Anterior-to-Posterior Migration of a Lumbar Disc Sequestration: Surgical Remarks and Technical Notes about a Tailored Microsurgical Discectomy

**DOI:** 10.1155/2017/1762047

**Published:** 2017-01-10

**Authors:** Alessandro Frati, Alessandro Pesce, Mauro Palmieri, Tommaso Vangelista, Riccardo Caruso, Maurizio Salvati, Antonino Raco

**Affiliations:** ^1^IRCCS Neuromed, Via Atinense 18, 86077 Pozzilli, Italy; ^2^Neurosurgery Division, A. O. Sant'Andrea, Via di Grottarossa 1035-1039, 00189 Roma, Italy; ^3^NESMOS Department, Sapienza University of Rome, Roma, Italy; ^4^Department of Neurology and Psychiatry-Neurosurgery, Sapienza University of Rome, Roma, Italy

## Abstract

Extrusion of disc material within the spinal canal complicates up to 28.6% of lumbar disc herniations. Due to the anatomical “corridors” created by the anterior midline septum and lateral membranes, relocation occurs with an anterior and anterolateral axial topography. Posterior migration is an extremely rare condition and anterior-to-posterior circumferential migration is an even rarer condition. Its radiological feature can be enigmatic and since, in more than 50% of cases, clinical onset is a hyperacute cauda equina syndrome, it may imply a difficult surgical decision in emergency settings. Surgery is the gold standard but when dealing with such huge sequestrations, standard microdiscectomy must be properly modified in order to minimize the risk of surgical trauma or traction on the nerve roots.

## 1. Introduction

A lumbar intervertebral disc sequestration derives from the extrusion of disc material within the spinal canal or the contiguous foramina through a laceration that involves two anatomical layers: the annulus fibrosus of the intervertebral disc and the posterior longitudinal ligament (PLL). It is included in the radiological presentation of a lumbar disc herniation in up to 28% of cases [1], but it usually migrates upward, downward, or in the foramina involving the anterior aspect of the spinal canal [1–6].

Posterior epidural migration is an absolutely rare entity, and up to now, it has been only exceptionally reported in the literature [1–10]. It usually presents as an emergency condition with severe neurological impairment such as cauda equina syndrome, and, therefore, since the radiological appearance can be unfamiliar even for a shrewd spine surgeon, this condition can imply a difficult surgical decision in emergency settings.

Moreover, since the syndromes associated with a such uncommon disc sequestration are a result of severe compressive polyradiculopathies [1, 5, 6, 10], a special tailored surgical technique is necessary, in order to avoid neurological adjunctive morbidity and improve neurological and clinical outcomes.

The aim of this work is therefore to report the most salient radiological and technical remarks of an exceptional spine surgery mockingbird: anterior-to-posterior lumbar disc sequestration.

## 2. Case Report

A 46-year-old female came to the Outpatient Neurosurgical Service of our institution complaining from a long-lasting history of lumbago radiating to the anterior side of the left lower limb. Pain was completely refractory to conservative treatments. She revealed progressively worsening hindrance in left flexion of the hip and numbness on the anterior aspect of the left thigh.

At physical examination, an obvious MRC 4-/5 strength deficit in the left flexion of the hip was evident with a superficial sensibilities impairment regarding cutaneous territories of L3 and L4 nerve roots. Left patellar reflex was abolished. Sphincter function was preserved.

**Figure 1:**
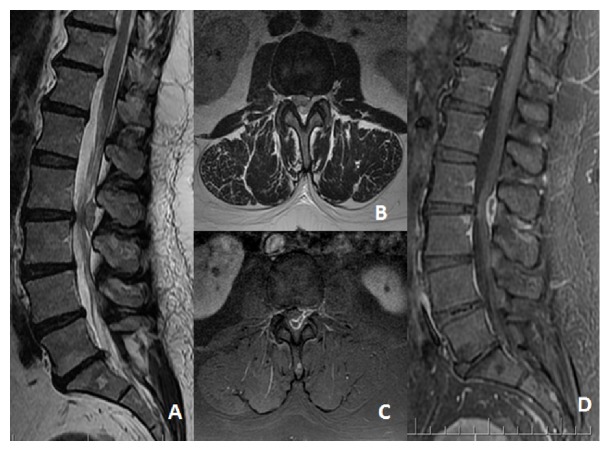
Preoperative MRI scan showing (A) sagittal, (B) axial T2W, (C) axial, and (D) sagittal gadolinium enhanced sequences showing the L2-L3 complete disc extrusion with an anterior-to-posterior circumferential course of the sequestered disc.

She underwent a standard lumbar spine MRI scan, which was performed with T1w, T2w, and STIR sequences and disclosed the presence of a huge L2-L3 disc sequestration relocated both caudally and from the anterior to the posterior aspect of the spinal canal with a circumferential epidural course causing severe compressive effects on L2, L3, and presumably L4 nerve roots (Figure 1).

Laboratory findings were within the normal range.

**Figure 2:**
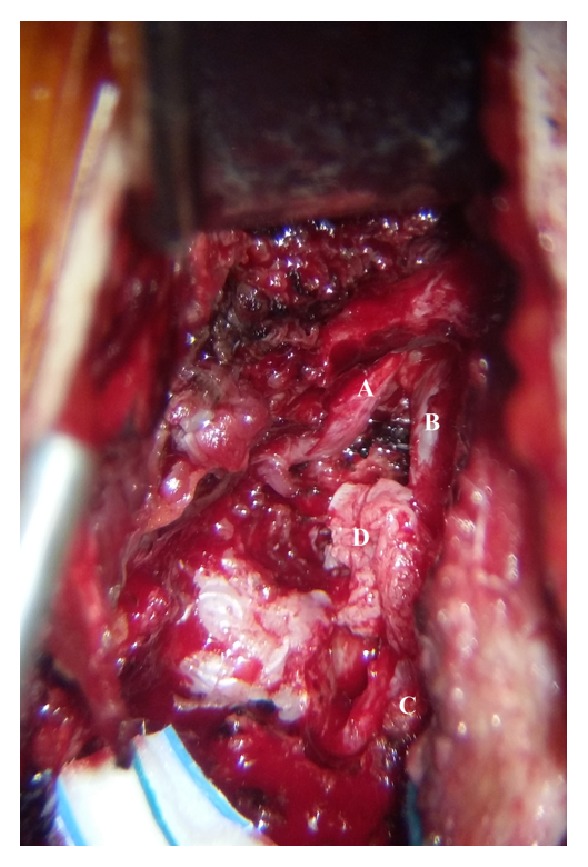
Intraoperative picture showing (A) L2 nerve root, (B) dural sac, (C) L3 nerve root, and (D) the posterior extruded segment.

She was referred to a surgical procedure of L2-L3 left partial interhemilaminectomy, wide arthrectomy, L2-L3 microdiscectomy and interspinous arthrodesis (realized with Aspen, Lanx, Inc., Broomfield, CO, USA), and L2 and L3 nerve roots adhesiolysis. Bony removal was intentionally generous because the sequestration was voluminous and the risk of performing incidental tractions or surgical traumas on nerve roots was high; furthermore, left L3 nerve root foraminotomy and adhesiolysis were difficult to perform with a standard approach for a simple L2-L3 discectomy (Figure 2). Moreover, the sequestered disc was surrounded, as usual, by an “inflammatory” tissue with tight adherence to the dural sac; therefore, maximizing the bony removal in such conditions decreased the risk of incidental durotomy and subsequent cerebrospinal fluid leakage. Despite the absence of an L2 radiculopathy, the reasons for a total L2-L3 arthrectomy lie in the necessity to

1. obtain full control on nerve roots and dural sac,
2. avoid surgical tractions on the nerve roots,
3. minimize the risk of incidental durotomy,
4. perform a satisfying nerve roots adhesiolysis.

**Figure 3:**
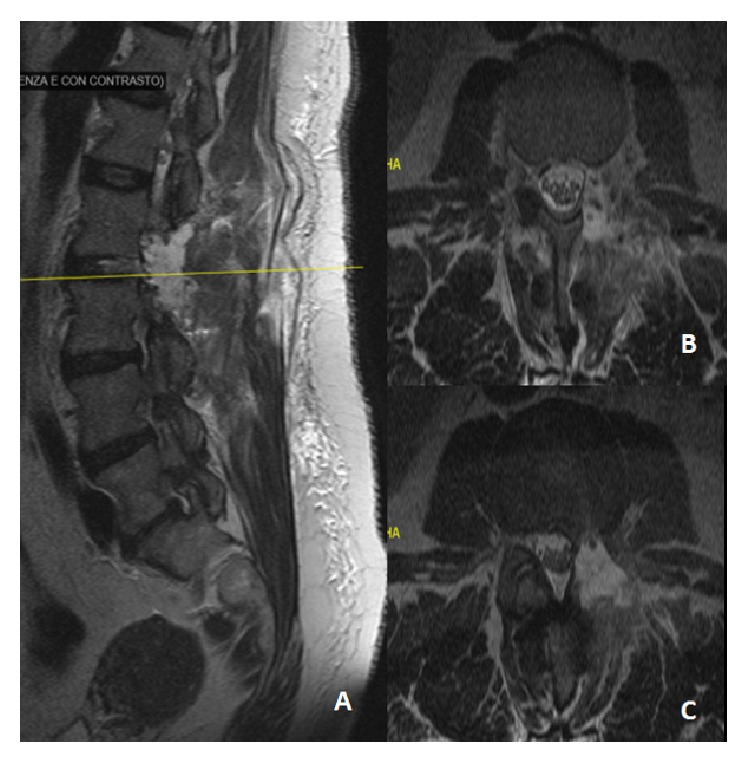
A postoperative MRI scan demonstrating complete resection of the sequestered disc material. Note the decompression of the dural sac and nerve root. L2-L3 arthrectomy and interspinous fixation at the same level were performed.

The postoperative course was uneventful. During the first postoperative day, a routine postoperative lumbar MRI scan was performed (Figure 3) and demonstrated a complete sequestrectomy and successful decompression of the nerve roots. At discharge, on the second postoperative day, the patient reported an obvious improvement of the preoperative deficits both for what concerns strength (back to 5/5) and sensibility with a clear-cut improvement of the numbness of the anterior aspect of the left lower limb. Patellar reflex was still absent.

## 3. Discussion

Intervertebral disc sequestration can complicate up to 28.6% of lumbar disc herniations [10]. The extruded disc usually migrates through anatomical corridors represented by the “midline septum” and “lateral membrane” [11, 12] and therefore posterior and contralateral migration are extremely rare eventualities. Posterior migration has been reported in about 70 cases in the literature, mostly involving L3-L4 [10], commonly presenting with polyradiculopathies and cauda equina syndrome [1–6].

The gold standard for diagnosis is a gadolinium enhanced lumbar spine MRI scan [1, 3, 9]. T1w, T2w, STIR, and T1w gadolinium enhanced sequences are mandatory to achieve diagnosis [1, 3]. In the vast majority of cases (up to 80% [1, 9–12]), the extruded disc material is iso/hypointense in T1w and hyperintense in T2w. The inflammatory changes in the local environment cause an increased fluid content in the extruded material which is responsible for T2w hyperintensity [2, 3]. Furthermore, inflammation brings about an increase of regional blood perfusion which is responsible for the STIR hyperintensity of the MRI scan; the central part of the sequestered disc is usually hypointense [6]. Contrast agent must be always used in order to rule out the most common topographical differential diagnosis [3]:

1. Metastatic epidural masses, which in general present a pronounced gadolinium enhancement, with history of malignancy.
2. Spinal epidural hematomas, which are isointense on T1w, without gadolinium enhancement.
3. Spinal epidural abscess, which may present very similar features if compared to disc sequestration; inflammatory involvement of the vertebral body and involvement of other intervertebral discs as well as coherent laboratory findings may provide important clues.
4. Miscellaneous space-occupying lesions, like synovial or radicular cists. Anatomical relationship with the facet joint or the nerve root is usually conclusive.

A conclusive diagnosis cannot rely solely on radiology: anamnesis and laboratory findings stand as cornerstones of the preoperative diagnosis: for example, in our case, there was no history of previous malignancies, coagulation disorders, or infections and laboratory findings did not disclose relevant findings, and preoperative MRI (Figure 1) demonstrated the facet joint integrity.

The current gold standard for the treatment of this condition is surgery [13]. A successful surgical procedure brings about fast and effective pain management and fast recovery and return to a normal independent life [13, 14]. Moreover, it dramatically reduces the need for anti-inflammatory drugs, thus minimizing the serious side effects of NSAIDs and opioids [14].

In cases like ours, sequestration was purely anterior-to-posterior and, in our experience, the standard microsurgical discectomy technique has to be slightly modified in order to increase the postoperative functional improvement: a wide unilateral arthrectomy and partial unilateral interlaminectomy with exposure of the dural sac, “exiting” and “transiting” nerve root, appears to be mandatory to avoid:

1. tractions or direct surgical traumas on nerve roots, which can be severely compressed or even endowed by disc material,
2. sequestration remnants, with risk of residual compressive effects on nerve roots (great sequestrations are not always easy to reach from smaller posterior spinal approaches),
3. incidental durotomies, with subsequent CSF leakage.

When a unilateral arthrectomy and partial interhemilaminectomy are performed, stability of the operated spinal FSU is usually not jeopardized [15]. Nevertheless, microdiscectomy induces an increased ROM in axial rotation and lateral bending [16]. However, conclusive data about the natural history and possible progression of an* “overlap” instability syndrome* of degenerative* plus* iatrogenic pathogenesis are still widely missing. Interspinous fusion devices can be employed in both stand-alone mode or supporting interbody fusion with cages [17–19]. The dynamic fusion achieved is biomechanically complete, such as other fixation devices [16, 19]. These implants reduce the load over facet joints, reduce intradiscal pressure [20, 21], and have been found to decrease abnormal iatrogenic postdiscectomy increase in ROM in flexion-extension and in axial rotation and lateral bending, thus helping to preserve spine stability in the long run.

## 4. Conclusion

Posterior migration of an extruded lumbar disc is a very rare entity and disc material continuity that realizes anterior-to-posterior migration of disc material is even rarer. It is not always easy to diagnose and in up to 51.35% of cases it presents as a neurological hyperacute syndrome. Possible surgical decisions may have to be made in unfavourable emergency settings. Radiological appearance is not always conclusive and patient history must be taken into account to perform a pertinent differential diagnosis. Generous bony removal is of great help in dissecting nerve roots from such prominent disc extrusion, thus preserving their function from the risk of iatrogenic damage caused by direct surgical traumas or incidental tractions.

## References

[1] Haddadi K., Qazvini H. R. G. (2016). Posterior epidural migration of a sequestrated lumbar disk fragment causing cauda equina syndrome in an old patient: a case report. *Clinical Medicine Insights: Case Reports*.

[2] Dösoğlu M., Is M., Gezen F., Ziyal M. (2001). Posterior epidural migration of a lumbar disc fragment causing cauda equina syndrome: case report and review of the relevant literature. *European Spine Journal*.

[3] Derincek A., Ozalay M., Sen O., Pourbagher A. (2009). Posterior epidural mass: can a posteriorly migrated lumbar disc fragment mimic tumour, haematoma or abscess?. *Acta Orthopaedica Belgica*.

[4] Akhaddar A., El-Asri A., Boucetta M. (2011). Posterior epidural migration of a lumbar disc fragment: a series of 6 cases: a review. *Journal of Neurosurgery: Spine*.

[5] Bonaroti E. A., Welch W. C. (1998). Posterior epidural migration of an extruded lumbar disc fragment causing cauda equina syndrome: clinical and magnetic resonance imaging evaluation. *Spine*.

[6] Chen C. Y., Chuang Y. L., Yao M. S., Chiu W. T., Chen C. L., Chan W. P. (2006). Posterior epidural migration of a sequestrated lumbar disk fragment: MR imaging findings. *American Journal of Neuroradiology*.

[7] Kim J.-S., Lee S.-H., Arbatti N. J. (2010). Dorsal extradural lumbar disc herniation causing cauda equina syndrome: a case report and review of literature. *Journal of Korean Neurosurgical Society*.

[8] Kuzeyli K., Çakr E., Usul H. (2003). Posterior epidural migration of lumbar disc fragments: report of three cases. *Spine*.

[9] Rahimizadeh A., Rahimizadeh A., Soufiani H. (2013). Posterior epidural migration of sequestered lumbar disc fragment causing cauda equina syndrome. *Coluna/ Columna*.

[10] Turan Y., Yilmaz T., Göçmez C. (2017). Posterior epidural migration of a sequestered lumbar intervertebral disc fragment. *Turkish Neurosurgery*.

[11] Schellinger D., Manz H. J., Vidic B. (1990). Disk fragment migration. *Radiology*.

[12] Elgamri A., Sami A., Aqqad A. (2009). Posterior migration of a lumbar disk herniation as a cause of cauda equina syndrome. *Journal de Radiologie*.

[13] Atlas S. J., Keller R. B., Wu Y. A., Deyo R. A., Singer D. E. (2005). Long-term outcomes of surgical and nonsurgical management of sciatica secondary to a lumbar disc herniation: 10 year results from the Maine lumbar spine study. *Spine*.

[14] Legrand E., Bouvard B., Audran M., Fournier D., Valat J. P. (2007). Sciatica from disk herniation: medical treatment or surgery?. *Joint Bone Spine*.

[15] Tsai K.-J., Murakami H., Lowery G. L., Hutton W. C. (2006). A biomechanical evaluation of an interspinous device (Coflex) used to stabilize the lumbar spine. *Journal of Surgical Orthopaedic Advances*.

[16] Parchi P. D., Evangelisti G., Vertuccio A. (2014). Biomechanics of interspinous devices. *BioMed Research International*.

[17] Gonzalez-Blohm S. A., Doulgeris J. J., Aghayev K., Lee W. E., Volkov A., Vrionis F. D. (2014). Biomechanical analysis of an interspinous fusion device as a stand-alone and as supplemental fixation to posterior expandable interbody cages in the lumbar spine: laboratory investigation. *Journal of Neurosurgery: Spine*.

[18] Karahalios D. G., Kaibara T., Porter R. W. (2010). Biomechanics of a lumbar interspinous anchor with anterior lumbar interbody fusion. *Journal of Neurosurgery: Spine*.

[19] Kaibara T., Karahalios D. G., Porter R. W. (2010). Biomechanics of a lumbar interspinous anchor with transforaminal lumbar interbody fixation. *World Neurosurgery*.

[20] Wilke H.-J., Drumm J., Häussler K., MacK C., Steudel W.-I., Kettler A. (2008). Biomechanical effect of different lumbar interspinous implants on flexibility and intradiscal pressure. *European Spine Journal*.

[21] Lazaro B. C. R., Brasiliense L. B. C., Sawa A. G. U. (2010). Biomechanics of a novel minimally invasive lumbar interspinous spacer: effects on kinematics, facet loads, and foramen height. *Neurosurgery*.

